# Fracture Toughness, Radiation Hardness, and Processibility of Polymers for Superconducting Magnets

**DOI:** 10.3390/polym16091287

**Published:** 2024-05-04

**Authors:** Anders Gaarud, Christian Scheuerlein, David Mate Parragh, Sébastien Clement, Jacob Bertsch, Cedric Urscheler, Roland Piccin, Federico Ravotti, Giuseppe Pezzullo, Ralf Lach

**Affiliations:** 1 European Organization for Nuclear Research (CERN), Esplanade des Particules 1, 1211 Geneva, Switzerlandjacob.bertsch@cern.ch (J.B.); federico.ravotti@cern.ch (F.R.);; 2 Polymer Service GmbH Merseburg, Geusaer Straße 81f, 06217 Merseburg, Germany

**Keywords:** epoxy resin, polyurethane, PMMA, PC, fracture toughness, superconducting magnet, irradiation, viscosity, FCC

## Abstract

High fracture toughness at cryogenic temperature and radiation hardness can be conflicting requirements for the resins for the impregnation of superconducting magnet coils. The fracture toughness of different epoxy-resin systems at room temperature (RT) and at 77 K was measured, and their toughness was compared with that determined for a polyurethane, polycarbonate (PC) and poly(methyl methacrylate) (PMMA). Among the epoxy resins tested in this study, the MY750 system has the highest 77 K fracture toughness of *K*_IC_ = 4.6 MPa√m, which is comparable to the *K*_IC_ of PMMA, which also exhibits linear elastic behaviour and unstable crack propagation. The polyurethane system tested has a much higher 77 K toughness than the epoxy resins, approaching the toughness of PC, which is known as one of the toughest polymer materials. CTD101K is the least performing in terms of fracture toughness. Despite this, it is used for the impregnation of large Nb_3_Sn coils for its good processing capabilities and relatively high radiation resistance. In this study, the fracture toughness of CTD101K was improved by adding the polyglycol flexibiliser Araldite DY040 as a fourth component. The different epoxy-resin systems were exposed to proton and gamma doses up to 38 MGy, and it was found that adding the DY040 flexibiliser to the CTD101K system did not significantly change the irradiation-induced ageing behaviour. The viscosity evolution of the uncured resin mix is not significantly changed when adding the DY040 flexibiliser, and at the processing temperature of 60 °C, the viscosity remains below 200 cP for more than 24 h. Therefore, the new resin referred to as POLAB Mix is now used for the impregnation of superconducting magnet coils.

## 1. Introduction

Nb_3_Sn magnet technology is used for the next generation of accelerator magnets that can reach magnetic fields beyond those achievable with Nb-Ti superconductors. The high luminosity upgrade for the Large Hadron Collider (HL-LHC) [1] is an important milestone, as it will be the first project where Nb_3_Sn magnets will be installed in a particle accelerator [2,3]. High-field accelerator magnets are also being developed for future particle-accelerator projects like the Future Circular Collider (FCC) [4,5] and for a muon collider [6].

Superconducting magnet coils made of brittle conductors like Nb_3_Sn are typically impregnated with an epoxy resin. The impregnation resin needs to provide dielectric insulation between the cables of a coil [7] and to fill the porosity between the cables that is present after coil winding. Filling the porosity in the coil with a resin is needed to avoid the mechanical stresses that are exerted on the coil during assembly and operation irreversibly degrading the conductor [8]. It has been suggested that cracking of the impregnation resin can generate superconductor instabilities [9,10].

To reach the full potential of high-field Nb_3_Sn magnets, typically a certain number of so-called training quenches of the virgin magnets is needed [11,12,13,14]. One goal of the development for future Nb_3_Sn accelerator magnets is to reduce the number of training quenches of the virgin magnets required to reach the operational magnetic field.

For their good mechanical, thermal, and chemical resistance properties, epoxy resins are widely used in many applications including electronic devices, adhesives, and surface coatings. However, the resistance of brittle epoxy resins against the initiation and the growth of cracks can be relatively poor [15].

The fracture toughness of the epoxy resins for coil impregnation is considered to be a relevant parameter influencing the magnet quench behaviour [9,16]. Micro-cracking of the epoxy-resin matrix in composites during thermal cycles to cryogenic temperatures is also a concern for their application in the storage of liquid hydrogen [17,18] or for liquid oxygen [19].

The impregnation of large magnet coils can last more than 10 h, and low viscosity and long pot life of the uncured resin mix are mandatory [20,21]. Moreover, the superconducting magnets in future particle accelerators will be exposed to high irradiation doses, which must not unacceptably degrade the functional properties of the impregnation system [22,23].

In the present study, we have determined the room temperature (RT) and 77 K fracture toughness of seven epoxy-resin systems and one polyurethane for possible application at cryogenic temperatures in superconducting devices. The fracture toughness of these epoxy resins was compared with that of the two widely used thermoplastics polycarbonate (PC) and poly(methyl methacrylate) (PMMA), PC being known as a particularly tough polymer [24].

For its good processing capabilities (low viscosity and long pot life) and relatively high radiation resistance [25] the CTD101K epoxy system is used for the impregnation of large Nb_3_Sn coils, and it is the baseline impregnation system for the HL-LHC superconducting magnets [1,2,3]. A drawback of the three-component CTD101K epoxy system is its relatively low fracture toughness compared to the fracture toughness of alternative resins studied for superconducting magnet coil impregnation [26]. The fracture toughness was improved by adding the polyglycol flexibiliser Araldite DY040 as a fourth component to the CTD101K epoxy-resin system.

To validate the modified resin system for superconducting coil impregnation, its processability and radiation hardness needed to be confirmed. The viscosity evolution of the uncured resin mixes was measured with and without a flexibiliser at the processing temperature of 60 °C. The effect of gamma and 24 GeV/c proton irradiation up to 38 MGy on the thermo-mechanical properties of the CTD101K three-component system and the four-component system with a flexibiliser is compared. The influence of the flexibiliser addition on the creep behaviour has been determined too.

## 2. Materials and Methods

### 2.1. The Samples

For the mechanical tests of the epoxy resins and polyurethane, 4 mm-thick plates were produced at the CERN polymer laboratory by vacuum impregnation [20]. Samples for fracture toughness, flexural, short-beam, and dynamic mechanical analysis (DMA) tests were cut out from these plates by water-jet cutting. Poly (methyl methacrylate) (PMMA) and polycarbonate (PC) samples were cut out of the 4 mm-thick extruded plates. All samples of a given material were produced from the same plate, thus eliminating uncertainties related to the sample production processes.

#### 2.1.1. CTD101K

CTD101K is a diglycidyl ether of bisphenol-A (DGEBA) with an anhydride hardener and accelerator-cured epoxy system [25]. The CTD101K three-component system consists of the epoxy resin CTD101K (100 parts by weight (pbw)), the hardener (90 pbw), and the accelerator (1.5 pbw). The curing-temperature cycle was 5 h–110 °C plus 16 h–125 °C post-curing, with a temperature ramp rate of 10 K per hour during heating.

#### 2.1.2. POLAB Mix (CTD101K+DY040)

CTD101K+DY040 sample plates were produced with 10 wt.% and with 20 wt.% Araldite DY040 relative to CTD101K epoxy resin. Araldite DY040 is a solvent-free, low-viscous hot-curing polyglycol flexibiliser [27], which is part of the Araldite F epoxy-resin system described below. First, the CTD101K epoxy resin and hardener were mixed and degassed, and then, DY040 was added. This mix was again degassed before the accelerator was added, and a final degassing was executed. The same curing-temperature cycle as the one for the CTD101K system was applied. In the following, the CTD101K+10 wt.% DY040 epoxy system is referred to as POLAB Mix.

#### 2.1.3. CEA Mix

The two-component resin system Huntsman Araldite^®^ CY192-1 (100 pbw) with the hardener Huntsman Aradur^®^ HY 918-1 (100 pbw) is used for the vacuum impregnation of motors and generators [28]. The Araldite CY192-1/Aradur HY 918-1 system has been used by CEA Saclay for the impregnation of Nb_3_Sn quadrupole coils [29] and is here referred to as CEA mix. The curing-temperature cycle comprised three isothermal plateaus of 24 h–80 °C, 34 h–120 °C, and 12 h–130 °C, as recommended by CEA for coil impregnation. In contrast, Huntsman recommends an additional 140 °C post curing step that has not been added in the present study.

#### 2.1.4. MSUT

The Araldite MY 740 (100 pbw)/Aradur HY 906 (90 pbw)/DY 062 (0.2 pbw) epoxy system, also referred to as MSUT Twente, has been developed by the University of Twente and was used for their impregnating high-field magnets. A 4 h–85 °C + 16 h–110 °C curing cycle has been applied [30].

#### 2.1.5. Araldite F

The Araldite F (100 pbw)/Aradur HY 905 (100 pbw)/flexibiliser DY 040 (10 pbw) epoxy system is used by the company ASG Superconductors S.p.A. for impregnation of magnet coils. The curing cycle was 10 h–100 °C + 48 h–135 °C, as recommended by ASG. To increase pot life for the impregnation of large coils, the Araldite F–Aradur HY 905–flexibiliser DY 040 system does not contain the accelerator DY 061 and filler that are recommended by Huntsman [31,32].

#### 2.1.6. MY750

The epoxy-resin system MY750 is composed of the liquid, unmodified, solvent-free bisphenol A resin Araldite MY750 (100 pbw) and the aliphatic polyamine hardener Aradur HY5922 (55 pbw) from Huntsman Corporation. MY750 is used for the impregnation of large coils for magnets and rotating machines. The curing cycle comprised 6 h–40 °C and 3 h–80 °C plateaus, as recommended by the supplier.

#### 2.1.7. Mix61

The so-called Mix61 epoxy-resin system has been added to this study as an epoxy-resin system exhibiting elastic–plastic deformation with stable crack propagation at RT. This epoxy system, also known as NHMFL 61, is a proprietary formulation of the National High-Magnetic Field Laboratory (NHMFL, Tallahassee, FL, USA). It is composed of a Bisphenol A diglycidyl ether-based resin (Part I), an amine-type hardener (Part II), an amine-type high molecular weight co-reactant (Part III), and a liquid low-molecular weight additive (Part IV) [33]. The process temperature is 60 °C, and the curing-temperature cycle was 16 h at 60 °C, followed by 24 h at 100 °C post-curing.

#### 2.1.8. Polyurethane

The Sika RE700+RE106 system is a transparent polyurethane for LED and electronic components protection. The two components Sika RE700 and RE106 are mixed in a 50/50 ratio and cured at RT. The gel time of the mix at 25 °C is 30 min [34].

#### 2.1.9. PMMA and PC

The epoxy resins and polyurethane fracture properties are compared with those of the widely used thermoplastics poly (methyl methacrylate) (PMMA) and polycarbonate (PC).

Tensile properties, density, hardness, glass transition temperatures (*T*_g_), and linear thermal expansion coefficients in the temperature range of 30–45 °C of the investigated materials are summarized in Table 1. Throughout this investigation the yield strength *σ*_y_ is defined as the 0.2% offset yield strength.

### 2.2. Fracture Toughness Measurements

Flexural fracture toughness tests were conducted in accordance with ISO 13586, “Plastics—Determination of fracture toughness (*G*_IC_ and *K*_IC_)—Linear elastic fracture mechanics (LEFM) approach” [39].

**Figure 1:**
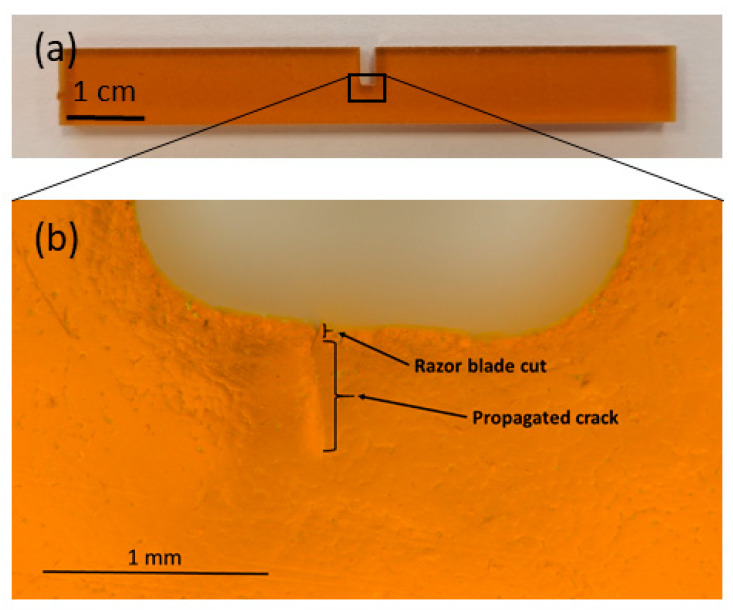
(**a**) SENB sample of CTD101K and (**b**) detailed view of the notch produced by water jet. The cut with a razor blade and the propagated crack prior to the fracture toughness measurement.

The critical stress-intensity factor (*K*_IC_) and the critical energy release rate (*G*_IC_) are derived from load–displacement curves measured in three-point bending of single-edge notched bending (SENB) specimens having nominal dimensions of 80 × 10 × 4 mm^3^. The samples have a 2 mm-wide and 5 mm-deep notch produced by a water jet. In all samples, a fine cut was added with a razor blade. A microscopic image of a typical crack produced with a razor blade in the notch of a CTD101K SENB sample is shown in Figure 1.

Three-point bending tests were executed in displacement control, with a force resolution of 0.17 N. Three-point bending was done with Ø = 10 mm loading supports and a Ø = 4 mm bending die. The span length was 40 mm (4× the nominal sample width). The measurements were conducted with a crosshead speed of 10 mm/min according to the standard.

**Figure 2:**
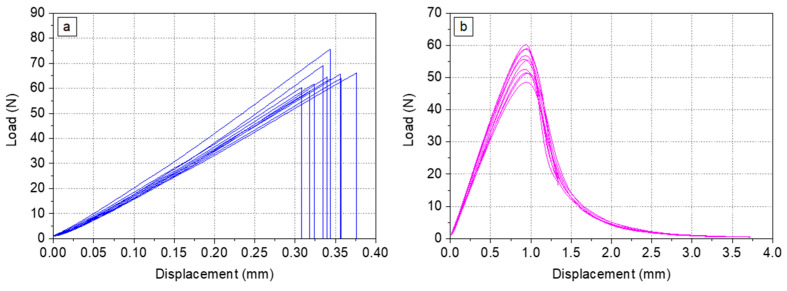
Load–displacement diagrams of SENB samples at RT for (**a**) MY750 and (**b**) Mix61.

Before the calculation of *K*_IC_ and *G*_IC_, it needs to be verified that the material represents linear elastic behaviour and that the crack propagation is unstable. As an example, load–displacement curves of the SENB samples made of MY750 (Figure 2a) and the corresponding fracture surface indicate linear elastic behaviour with unstable crack propagation. In contrast, the Mix61 load–displacement curves (Figure 2b) and fracture surface are indicative for elastic–plastic deformation with stable crack propagation.

**Figure 3:**
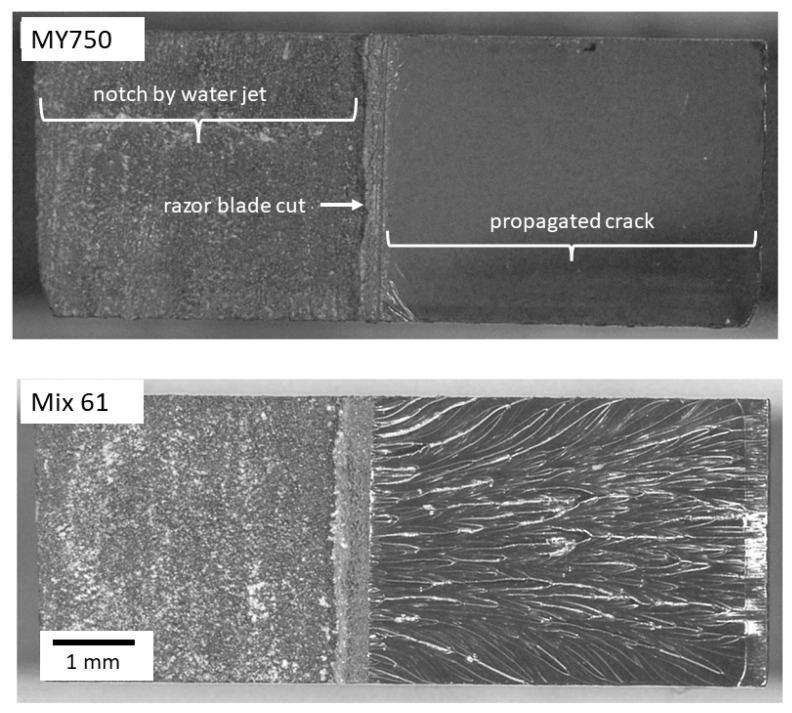
Fracture surface of SENB samples MY750 with unstable and Mix61 with stable crack propagation. The micrographs were obtained by using a reflected-light microscope (digital microscope VHX-500F from the company Keyence).

Typical fracture surfaces representing unstable crack propagation (MY750) and stable crack propagation (Mix61) are shown in Figure 3.

According to ISO 13586, for linear elastic fracture mechanics in plane-strain conditions around the crack tip, the fracture mechanics parameter critical stress-intensity factor (*K*_IC_) can be calculated from the load at crack growth initiation and from the sample and initial crack dimensions. The energy released per crack surface area may be expressed by the fracture mechanics parameter critical energy-release rate (*G*_IC_).

For fully linear elastic behaviour, as shown in Figure 2a, the maximum load *F*_max_ is used to calculate the provisional fracture toughness *K*_Q_. Otherwise, as shown in Figure 2b, the load *F*_Q_ at the interaction between the straight line related to the 95%-elastic slope and the load–displacement curve is taken for calculation of *K*_Q_.

For the calculation of *G*_IC_, the uncorrected work at break (up to *F*_max_) or up to *F*_Q_ was used.

To ensure plane-strain conditions around the crack tip, it needs to be verified that the provisional fracture toughness result is geometry independent according to the following sample size criteria, as described in ISO 13586:

(1) $$\mathrm{thickness}\;(h)\;>\;2.5\;\times \;\bar{r}$$

(2) $$\mathrm{crack}\;\mathrm{length}\;(a)\;>\;2.5\;\times \;\bar{r}$$

(3) $$\mathrm{ligament}\;\mathrm{width}\;(w-a)\;>\;2.5\;\times \;\bar{r}$$

The characteristic length $\bar{r}$ can be calculated by the provisional fracture toughness *K*_Q_ and the yield strength of the material (*σ*_y_) according to Equation (4):

(4) $$\bar{r}=\frac{K_{Q}^{2}}{\sigma_{y}^{2}}$$

The above size criteria assessment was investigated for metallic materials first and used for polymer materials later also. However, detailed experimental investigations on polymers (including epoxy resins) have shown that the constant factor of 2.5 may be replaced by 3466 × *K*_Q_^−1.73^ (*K*_Q_ in MPa mm^1/2^) [40].

### 2.3. Dynamic Mechanical Analysis (DMA)

The storage modulus (*G*′) and loss modulus (*G*″) were recorded during temperature sweeps with a Dynamical Mechanical Analyser MCR702e from Anton Paar, Graz, Austria. Glass transition temperatures have been derived according to ASTM D 4065, DIN EN ISO 11357 [41,42], at a frequency of 1 Hz and a temperature ramp of 2 K/min. During the temperature sweeps, the thermal expansion of the samples has been measured as described in [23]. Irradiation-induced changes of the rubbery shear modulus (*G*′_rubbery_) have been determined as a measure of the molecular weight between the crosslinks and the crosslink density [43,44,45].

### 2.4. Viscosity Measurements

Viscosity measurements were performed with a Lamy Rheology CP2000 plus cone–plate viscometer operated in speed control.

### 2.5. Gamma Irradiation

Gamma irradiation was performed with a ^60^Co source in ambient air at a temperature of 20–25 °C, with an approximate dose rate of 2 kGy/h. More details about the irradiation procedures and the irradiation hardness of the epoxy resins in this study can be found in [23].

### 2.6. Proton Irradiation

Irradiations with 24 GeV/c protons have been performed at the CERN IRRAD facility, with an average proton fluence of about 1.4 × 10^16^ p/cm^2^ per week, corresponding to a dose of about 4 MGy per week. Irradiation experiments in IRRAD are performed with a pulsed beam delivering about 5 × 10^11^ protons per pulse of ~400 ms length. During one year of operation at IRRAD, a dose of about 35 MGy can be accumulated. More details about the IRRAD facility and the sample dosimetry measurements can be found in reference [46].

## 3. Results

### 3.1. RT and 77 K Fracture Toughness of CTD101K with Different Flexibiliser Content

**Figure 4:**
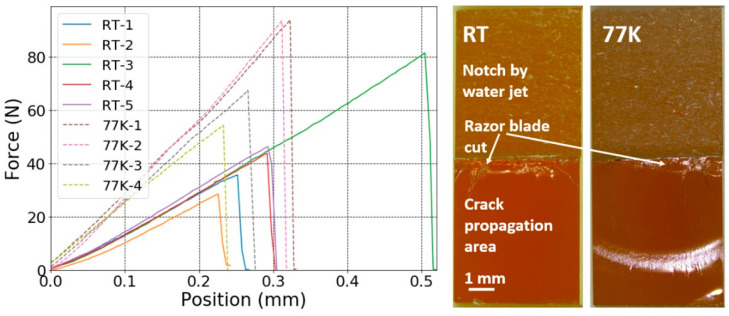
Load–displacement curves of CTD101K+20 wt.% DY040 specimens (left) and typical fracture surface images (right) at RT and 77 K.

The load–displacement curves of the notched SENB CTD101K+20 wt.% DY040 samples acquired at RT and 77 K and the corresponding images of typical fracture surfaces are presented in Figure 4. From the load–displacement curves and fracture surfaces, it can be seen that, like the three-component CTD101K, the four-component systems with 10 wt.% and 20 wt.% DY040 flexibiliser exhibit linear elastic behaviour and unstable crack propagation criteria.

**Figure 5:**
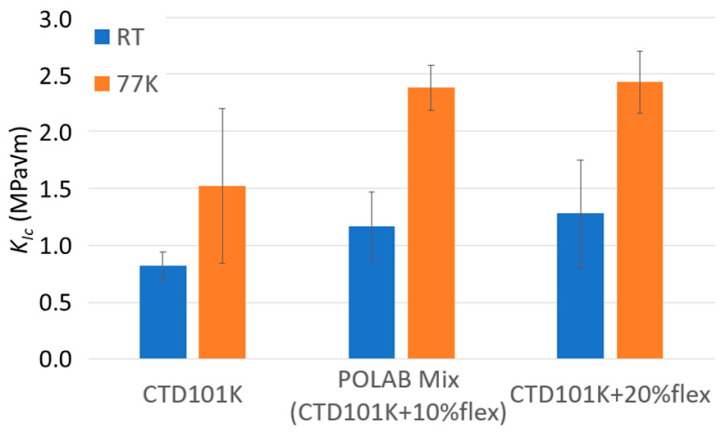
Comparison of the critical stress-intensity factor *K*_IC_ at RT and 77 K of CTD101K, POLAB Mix (CTD101K+10 wt.% DY040) and CTD101K+20 wt.% DY040. Error bars: ±1σ.

*K*_IC_ values determined for the CTD101K systems with and without DY040 flexibiliser are compared in Figure 5. The fracture toughness of the CTD101K resin system is significantly improved by adding 10 wt.% flexibiliser DY040 (POLAB Mix). The effect is particularly strong at cryogenic temperature; the fracture toughness at 77 K can be increased from *K*_IC_ = 1.5 MPa√m to *K*_IC_ = 2.4 MPa√m. There is no significant *K*_IC_ difference between the systems with 10% and with 20% flexibiliser.

### 3.2. RT and 77 K Fracture Toughness of the Polymers Exhibiting Linear Elastic Behaviours and Unstable Crack Propagation

**Figure 6:**
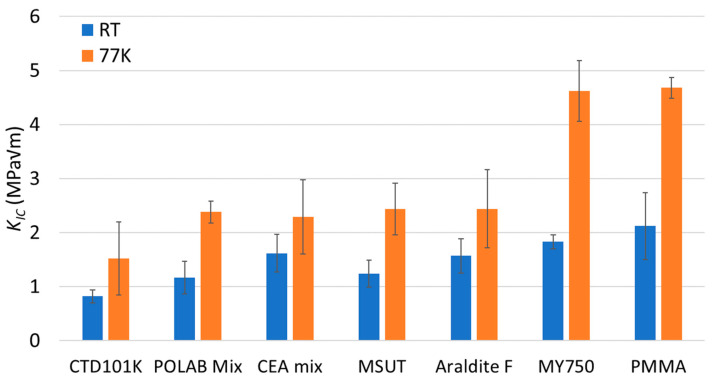
Fracture toughness parameters critical stress-intensity factor (*K*_IC_) of CTD101K, POLAB Mix, MY750, CEA mix, MSUT, Araldite F, and PMMA at RT and at 77 K. Error bars: ±1σ.

In Figure 6, the RT and 77 K *K*_IC_ values of the different epoxy-resin systems and the PMMA are compared. All materials presented in Figure 6 exhibit linear elastic behaviour and unstable crack propagation. The geometric size criteria for plane-strain conditions are fulfilled too (see Table 2). Thus, the *K*_IC_ and *G*_IC_ values of the materials presented in Figure 6 allow for a comparison of the fracture toughness.

The *K*_IC_ of POLAB Mix is comparable to that of the CEA mix, MSUT, and Araldite F epoxy systems. These systems have the highest fracture toughness of the epoxy resins of this study that can be used for the impregnation of large coils.

The MY750 system’s fracture toughness is comparatively higher than that of the other epoxy resins tested and comparable to that of PMMA. However, the pot life of MY750 is so short that it cannot be processed in long magnet coils, and its radiation resistance is poor [47].

### 3.3. Fracture Toughness of Polyurethane and PC

**Figure 7:**
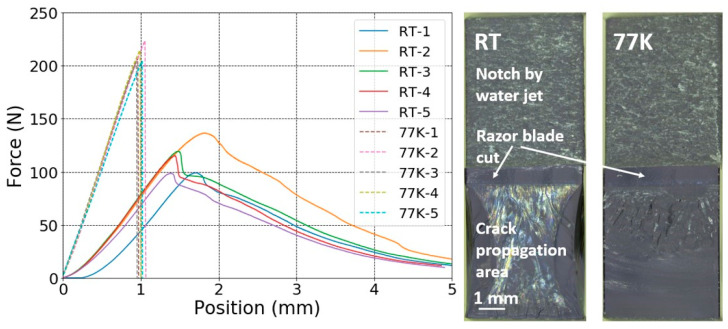
Load–displacement curves of PC specimens (**left**) and typical fracture surface images (**right**) at RT and 77 K.

The load–displacement curves acquired at RT and at 77 K of the PC notched SENB samples, and the typical RT and 77 K fracture surfaces are presented in Figure 7. At RT PC yields plastically and at 77 K, it exhibits linear elastic behaviour. The notched SENB sample fracture surfaces indicate stable crack propagation at RT and at 77 K. Thus, the resistance to fracture is underestimated by applying the fracture toughness parameter *K*_Q_ (critical stress-intensity factor), and the fracture toughness parameter *G*_Q_ (critical energy-release rate) is more appropriate to compare the toughness.

**Figure 8:**
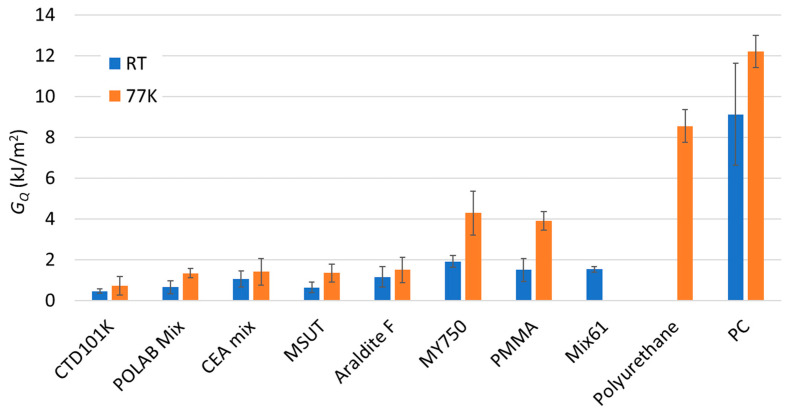
Comparison of critical energy-release rate (*G*_Q_) of CTD101K, POLAB Mix, MY750, CEA mix, MSUT, Araldite F, Polyurethane, PMMA, and PC at RT and at 77 K. Error bars: ±1σ.

In Figure 8, the *G*_Q_ values of the different epoxy-resin systems are compared with those measured for polyurethane, PMMA, and PC. Polyurethane (which is flexible at RT), has outstandingly high fracture properties at 77 K, which are only exceeded by those of PC, which is known for its very high fracture toughness.

Table 2 summarises the *K*_Q_ and *G*_Q_ derived and the material and sample parameters that are needed to verify plane-strain conditions around the crack tip. As a conservative estimate at 77 K, a yield stress of 100 MPa has been used to calculate the 2.5$\bar{r}$ values for all materials. The geometric size criteria for the plane-strain conditions are met for all epoxy-resin systems and test conditions studied. Thus, for these materials, *K*_Q_ and *G*_Q_ correspond with the critical stress-intensity factor (*K*_IC_) and critical energy-release rate (*G*_IC_), respectively.

For the MY750 and PMMA tested at 77 K, the size criterion of 2.5$\bar{r}\;$ slightly exceeds the ligament width. Larger Mix 61, polyurethane, and PC samples would be needed to fulfil the geometric size criteria for plane-strain conditions.

### 3.4. RT Tensile Stress–Strain Behaviour, Creep, and Stress Relaxation of CTD101K and POLAB Mix

**Figure 9:**
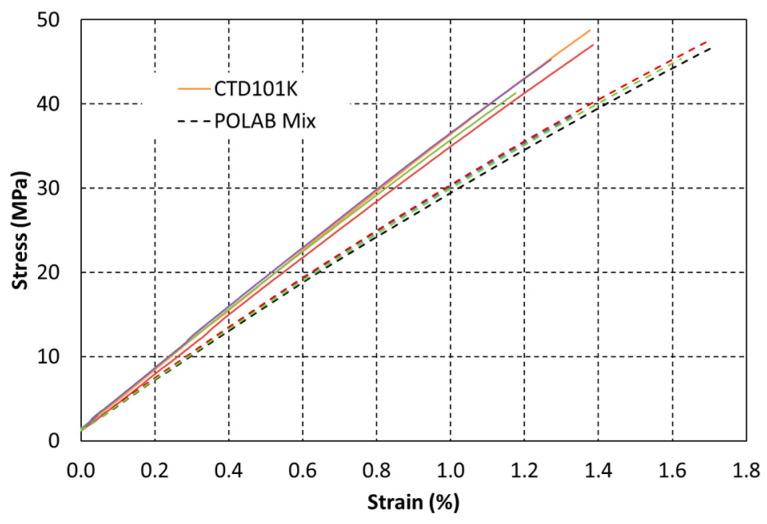
Uniaxial tensile stress–strain curves of CTD101K (continuous lines) and POLAB Mix (dashed lines) acquired at 23 °C according to [37].

The uniaxial tensile stress vs. strain curves of CTD101K and POLAB Mix are compared in Figure 9. CTD101K exhibits linear elastic behaviour up to its fracture stress of about 45 MPa. Unlike the CTD101K three-component system, the CTD101K four-component system with the addition of the flexibiliser DY040 exhibits some plasticity, and the strain at the fracture is increased.

**Figure 10:**
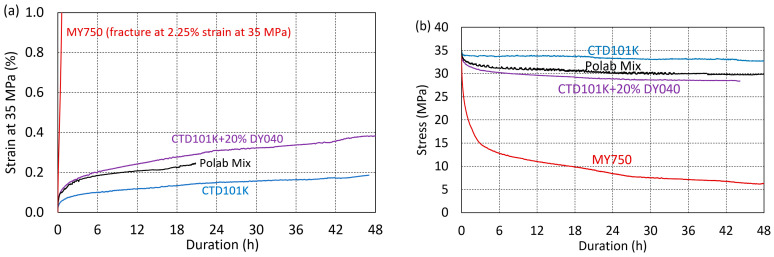
Comparison of CTD101K, POLAB Mix, CTD101K+20 wt.% DY040, and MY750 (**a**) tensile strain as a function of duration at constant stress of 35 MPa (creep) and (**b**) tensile stress as a function of duration at constant strain applied at initially 35 MPa (stress relaxation).

The effect of the flexibiliser addition on creep and stress relaxation has been determined under uniaxial tensile loading at ambient temperature (Figure 10). Pure CTD101K exhibits a comparatively small stress relaxation behaviour, as it loses only ~6% of its pre-stress after 48 h. The addition of 10% and 20% DY040 flexibiliser to CTD101K increases the stress relaxation slightly. The strong creep and stress relaxation behaviour of the tough MY750 resin is shown for comparison.

### 3.5. Viscosity of the Uncured Epoxy-Resin Mix

**Figure 11:**
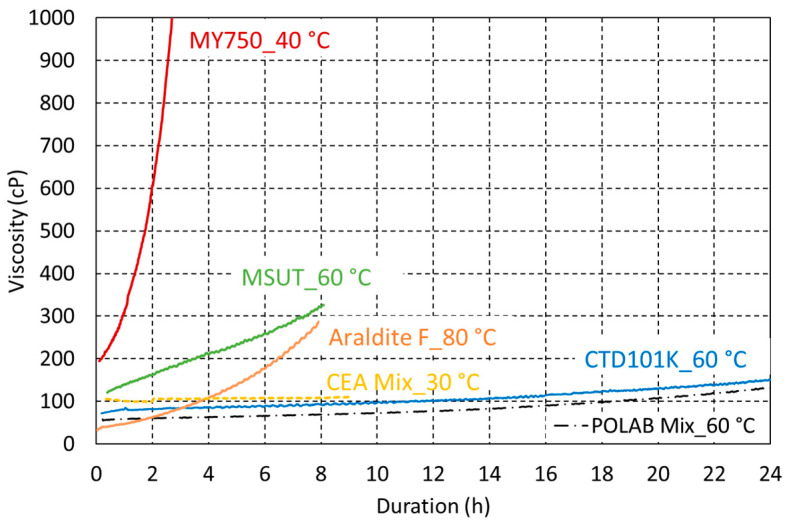
Comparison of the viscosity evolution of CTD101K and POLAB Mix as a function of duration at the processing temperature of 60 °C with the viscosity evolution of CEA Mix at 30 °C, MSUT at 60 °C, Araldite F (without accelerator) at 80 °C, and MY750 at 40 °C.

The viscosity evolutions of the uncured POLAB Mix and CTD101K epoxy resin at the processing temperature of 60 °C are compared in Figure 11. The excellent processibility of CTD101K is not affected by the addition of 10 wt.% flexibiliser DY040. For comparison, the viscosity evolutions of Araldite F, MSUT, and MY750 at their respective processing temperatures are presented too.

Typically, the viscosity of the impregnation resin should not exceed 500 cP during the impregnation process. Thus, the resins MY740, Araldite F (without accelerator), CTD101K, and POLAB Mix can be used for coil impregnation processes lasting for several hours. The addition of the flexibiliser does not significantly change the viscosity of the CTD101K system, and the POLAB Mix has a similarly very low viscosity, offering excellent processability. The MY750 viscosity evolution does not allow the vacuum impregnation of large magnet coils, for which the impregnation process can last up to 10 h.

### 3.6. Radiation-Induced Changes of Viscoelastic Properties

The effect of ionizing irradiation on crosslinking and chain scission has been derived from the storage modulus *G*′(*T*) and loss modulus *G*″(*T*) evolutions as described in detail in [23,47].

**Figure 12:**
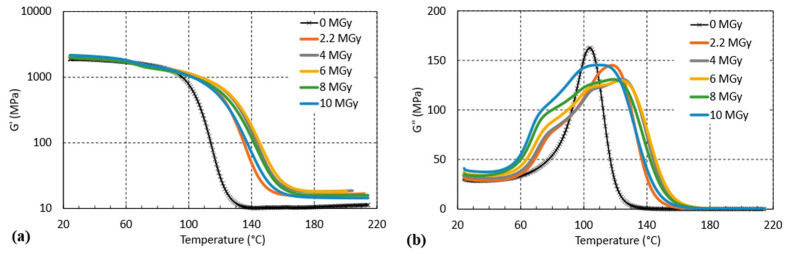
(**a**) *G*′(T) and (**b**) *G*″(T) of CTD101K+20% DY040 after different absorbed gamma doses.

Figure 12 shows the *G*′(*T*) and *G*″(*T*) evolutions of the CTD101K+20% DY040 system after increasing the ^60^Co gamma irradiation doses, which reveals a competition between crosslinking and chain scission. On the one hand, this can be seen by the distinct shift of the glass transition-induced drop of *G*′ to higher temperatures up to 6 MGy and the shift of this drop back to lower temperatures at higher doses.

Moreover, a pronounced broadening of the glass transition range as seen in *G*″ (Figure 12b) and splitting in a lower-temperature branch (shoulder) and a higher-temperature branch (*G*″ maximum) emphasise this competition. The lower-temperature branch presumably corresponds to a lower crosslink density fraction of the polymer produced by chain scission, whereas the probably crosslinking-induced higher-temperature branch is shifting to higher temperatures, up to a dose of 6 MGy first and shifting to lower temperatures at higher doses.

**Figure 13:**
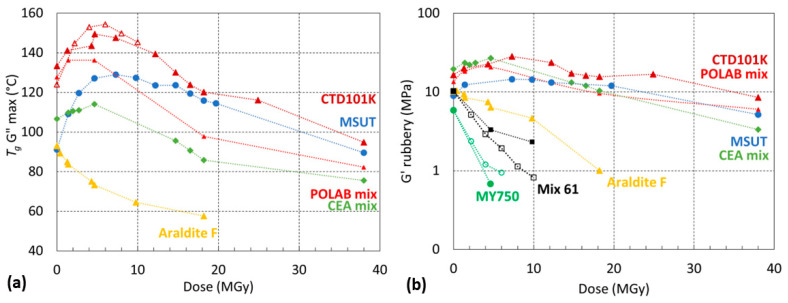
(**a**) *T*_g_ G″ max and (**b**) *G*′_rubber*y*_ as a function of absorbed dose (^60^Co gamma in open symbols or 24 GeV/c proton irradiation in full symbols) in ambient air. The dashed lines are a guide to the eye.

Figure 13 compares the *T*_g_ and *G*′_rubbery_ evolutions of the seven epoxy resins as a function of the absorbed proton dose. The CTD101K, POLAB Mix, MSUT, and CEA Mix maintain a comparatively high *G*′_rubbery_ of up to 38 MGy. The Araldite F epoxy system degrades at lower doses. The highest chain scission rates are observed for MY750 and Mix 61, which are not suitable for use in high-dose environments.

### 3.7. Radiation-Induced Changes of RT Mechanical Properties

**Figure 14:**
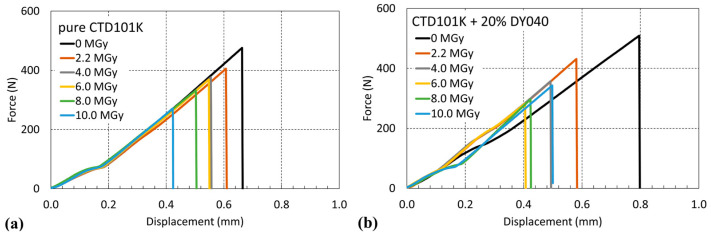
Force–displacement curves in short-beam test configuration of (**a**) CTD101K and (**b**) CTD101K+20% DY040 before and after ^60^Co irradiation in ambient air up to 10 MGy.

The three-point bending force–displacement results in the short-beam test configuration [48] of the three-component CTD101K and the four-component CTD101K+20%DY040 flexibiliser systems after different gamma doses are compared in Figure 14.

**Figure 15:**
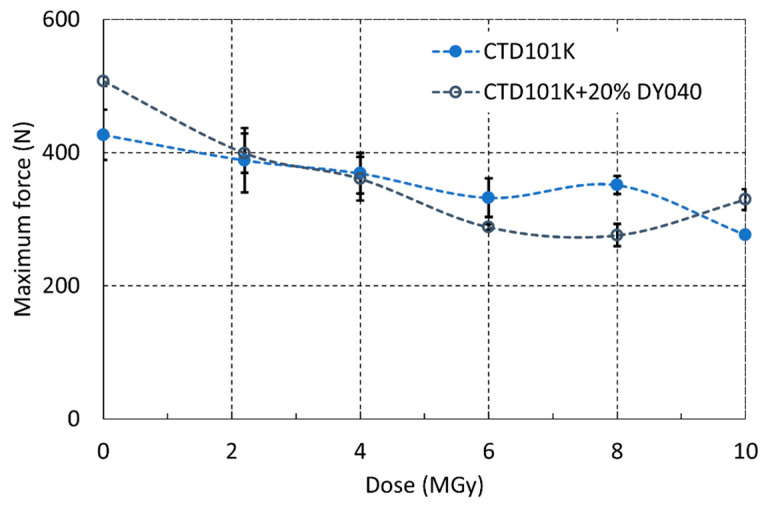
Effect of ^60^Co gamma irradiation at ambient temperature on CTD101K and CTD101K+20% DY040 force at fracture during 3-point bending test in short-beam configuration.

In Figure 15 the short-beam strength of the CTD101K and CTD101K+20% DY040 epoxy systems is plotted as a function of absorbed gamma ray dose. For each material and dose step, three samples have been tested. There is no significant difference in the effect of irradiation on the short-beam strength of both systems.

The thermoplastics PMMA and PC strongly degrade at comparatively low doses (<1 MGy), and the radiation hardness of PC somewhat exceeds that of PMMA [49]. The irradiation-induced colour changes of PMMA up to about 100 kGy can be exploited for dosimetry measurements [50,51].

**Figure 16:**
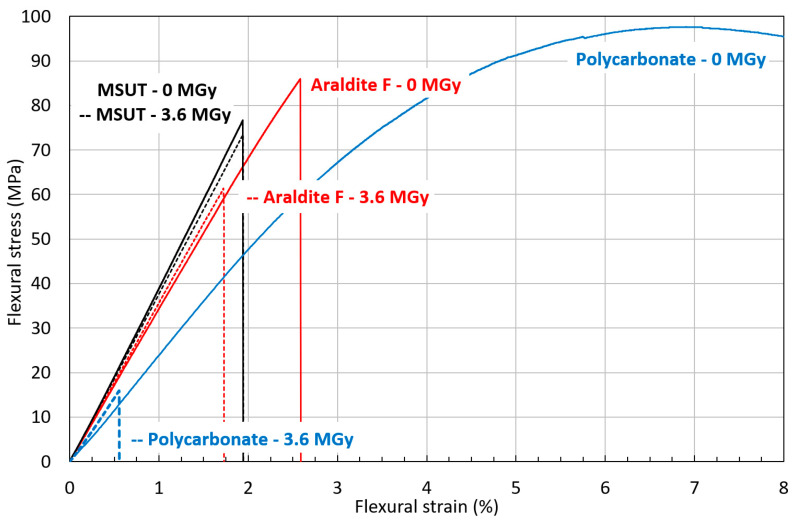
Comparison of RT flexural stress–strain curves of MSUT, Araldite F, and PC before and after 3.6 MGy gamma irradiation. The irradiated material stress–strain curves are represented by dashed lines.

Flexural stress–strain curves of MSUT, Araldite F, and PC before and after irradiation to 3.6 MGy are presented in Figure 16. The epoxy-resin system MSUT shows almost no difference in flexural properties before and after irradiation to 3.6 MGy. The epoxy-resin system Araldite F has a significantly lower flexural strength after irradiation to 3.6 MGy. The thermoplastic PC has outstanding flexural properties in the unirradiated condition but dramatically degrades after irradiation to 3.6 MGy, with a reduction of flexural strain at maximum stress from 6.7% to 0.6%. After 3.6 MGy, PMMA does not keep any mechanical strength.

## 4. Discussion and Conclusions

The fracture toughness of the epoxy resins used for the impregnation of superconducting magnet coils is a parameter that can influence the magnet quench behaviour, and a higher fracture toughness may reduce the number of quenches that are needed to achieve the required magnetic fields [26].

For polymers exhibiting unstable crack propagation and linear elastic behaviour, the fracture toughness can be compared based on linear elastic fracture mechanics [39]. In the present study, this is the case for CTD101K, POLAB Mix, CTD101K+20% DY040, MSUT, CEA Mix, Araldite F, MY750, and PMMA. The fracture toughness of these materials can be expressed in terms of the critical stress-intensity factor (*K*_IC_) [52].

MY750 has the highest 77 K fracture toughness of the examined epoxy-resin systems [26]. The MY750 *K*_IC_ is comparable to that of PMMA. The three-component CTD101K resin exhibits the lowest *K*_IC_ of all materials in the present study. The fracture toughness of epoxy resins can be improved in different ways, including the addition of rubber [53,54], or the addition of nano-powders [55,56]. Here we have improved the CTD101K fracture toughness by adding a polyglycol flexibiliser. The *K*_IC_ results of the CTD101K system without and with the flexibiliser confirm that the toughness of the CTD101K resin system can be improved by adding 10 wt.% flexibiliser DY040. Further increasing the flexibiliser content to 20 wt.% does not significantly change *K*_IC_ (Figure 5). The *K*_IC_ value of the POLAB Mix epoxy-resin system (CTD101K with 10 wt.% DY040) is comparable to that of the MSUT, CEA mix, and Araldite F epoxy-resin systems.

To validate the POLAB Mix epoxy-resin system for use in superconducting magnet coils, the viscosity evolution of the uncured resin mixes at the respective processing temperatures was measured. The long pot life and very low viscosity are not affected by the addition of the flexibiliser, enabling the impregnation of large coils lasting many hours. The viscosity evolutions of the Araldite F three-component system without the accelerator and of the MSUT system at their respective processing temperatures also enable impregnation processes lasting up to about 10 h. Because of its comparatively short pot life (Figure 11), MY750 cannot be used for the vacuum impregnation of large magnet coils.

Another concern of the flexibiliser addition was that it might reduce the good irradiation hardness of the CTD101K three-component system. Therefore, we have tested both systems after different gamma-ray and proton doses. The effect of gamma and proton irradiation on *T*_g_ is similar for both systems. Irradiation up to 6 MGy increases *T*_g_ due to the formation of new crosslinks. Further increasing the irradiation dose causes a *T*_g_ reduction, because the chain scission rate exceeds the rate with which links are formed. The comparison of the short-beam test results confirms that the ageing of both resin systems occurs at a similar rate.

Measurement of *T*_g_ and *G*′_rubbery_ evolution as a function of 24 GeV/c proton dose shows that CTD101K, POLAB Mix, MSUT, and CEA Mix exhibit similar chain scission rates, and maintain some mechanical strength even up to a very high dose of 38 MGy. The Araldite F system chain scission rate is comparatively higher. Mix61 and MY750 are not suitable for use in high irradiation environments [47].

Stress relaxation of the POLAB Mix under uniaxial tensile loading at RT is somewhat increased with respect to that of the very brittle three-component CTD101K system. The increased stress relaxation might be beneficial to reduce stress concentrations due to geometrical imperfections of the coils and other magnet constituents, but it may also contribute to an unwanted loss of pre-stress in the magnets.

Of the epoxy-resin systems studied here, POLAB Mix, CEA Mix, and MSUT offer the best compromise of the conflicting requirements for improving fracture toughness and irradiation hardness for their application in superconducting magnets. For its improved fracture toughness, combined with excellent processability and good radiation resistance, POLAB Mix is now used at CERN for the impregnation of superconducting magnet coils like Canted–Cosine–Theta dipoles [57].

Fracture toughness measurements at 4.2 K are in preparation to verify if the epoxy resin 77 K fracture toughness is relevant for predicting the fracture toughness during superconducting magnet operation in liquid helium [58].

## Figures and Tables

**Table 1:** Densities (*ρ*) [35], mechanical parameters at 23 °C, and thermal parameters of the materials investigated. Ball indentation hardness (*HB*) according to [36], tensile modulus (*E*), yield stress (*σ*_y_) ultimate stress (*σ*_m_), and strain at ultimate stress (*ε*_B_) according to [37]. *T*_g_ is derived from *G*′_onset_, *G*″_max,_ and tan *δ*_max_ (*G*′, *G*″—storage and loss modulus from DMA under torsion, tan *δ* = G″/G′), and the linear thermal expansion coefficient (*α*) is determined in the temperature range 30–45 °C. * *E*′, *E*″, and tan *δ*_max_ at a heating rate of 4 K/min (*E*′, *E*″—storage modulus and loss modulus from DMA under tension, tan *δ* = *E*″/*E*′). ** Shore A hardness = 74.5 ± 0.4, measured according to [38]. Not measured (n.m.).

Material	*ρ* (g/cm^3^)	*HB* (MPa)	*E* (GPa)	*σ*_y_ (MPa)	*σ*_M_ (MPa)	*ε*_B_ (%)	*T*_g_ (°C)			*α* (×10^−6^ K^−1^)
							*G*′_onset_	*G*″_max_	tan *δ*_max_	
CTD101K	1.22	217 ± 5	3.53 ± 0.08	-	46 ± 3.2	1.3 ± 0.1	118	122	140	60
POLAB Mix	1.21	199 ± 8	3.07 ± 0.03	-	45 ± 3.9	1.6 ± 0.2	124	128	140	68
CEA mix	1.27	213 ± 14	3.86 ± 0.13	-	53 ± 5.5	1.5 ± 0.2	100	107	113	56
MSUT	1.23	245 ± 12	3.59 ± 0.07	-	45 ± 5.6	1.3 ± 0.2	88	91	107	60
Araldite F	1.22	199 ± 5	3.30 ± 0.06	54	59 ± 5.7	2.0 ± 0.3	91	93	105	55
MY750	1.15	126 ± 4	3.05 ± 0.07	46	60 ± 0.4	2.8 ± 0.1	49	51	55	62
Mix61	1.14	47.5 ± 2	0.67 ± 0.07	13	20 ± 0.4	19 ± 0.4	−38 *	n.m.	73	144
Polyurethane	1.13	**	~0.08	n.m.	~2.4	~30	−16 *	−11 *	1.0 *	n.m.
PMMA	1.2	202 ± 5	3.25 ± 0.14	51	61 ± 2.0	2.3 ± 0.1	115	118	133	n.m.
PC	1.20	127 ± 3	2.35	34	60	~5	150	152	158	63

**Table 2:** Yield stress or tensile strength (*σ*_y_, *σ*_M_), *K*_Q_, and *G*_Q_ according to [39] at 23 °C and at 77 K. Sample thickness (*h*), sample width (*w*), and ligament width (*w*–*a*). For plane-strain conditions to be fulfilled, the sample dimensions must exceed 2.5 times the characteristic length (2.5$\bar{r}$). For Araldite F, MY750, PMMA, Mix 61, and PC, the yield stress values *σ*_y_ (0.2% offset yield strength) are used for the calculation of 2.5$\bar{r}$. The other epoxy resins fractured before the 0.2% offset yield strength was reached, and the tensile strength *σ*_M_ *_m_* was used (values in italic). * The 77 K yield strength values are estimated. ^#^ For Mix 61 and PC the fracture mechanics values do not fulfil plane-strain conditions.

Material	σ_y,_ σ_M_ (MPa)	*K*_Q_ (MPa√m)	*G*_Q_ (kJ/m^2^)	*h* (mm)	*a* (mm)	*w*–*a* (mm)	$2.5\bar{r}$
	RT						
CTD101K	*46*	0.82 ± 0.12	0.45 ± 0.10	4.03	5.50	4.54	0.8
POLAB Mix	*45*	1.17 ± 0.30	0.65 ± 0.32	3.95	5.26	4.83	1.7
CTD101K+20% DY040	*40*	1.28 ± 0.47	0.79 ± 0.58	4.22	5.28	4.79	2.6
CEA mix	*53*	1.62 ± 0.35	1.05 ± 0.40	3.83	4.31	5.78	2.3
MSUT	*46*	1.24 ± 0.25	0.63 ± 0.26	3.93	5.25	4.84	1.8
Araldite F	54	1.57 ± 0.32	1.14 ± 0.50	3.89	5.13	4.96	2.1
MY750	46	1.83 ± 0.13	1.91 ± 0.30	4.00	5.48	4.56	4.0
PMMA	51	2.12 ± 0.62	1.50 ± 0.56	3.69	5.69	4.35	4.3
Mix 61	13	1.17 ± 0.05	1.52 ± 0.13	3.90	4.60	5.36	20 ^#^
PC	54	3.51 ± 0.41	9.12 ± 2.49	3.97	5.56	4.64	27 ^#^
	77 K						
CTD101K	100 *	1.52 ± 0.68	0.72 ± 0.46	4.00	5.34	4.70	0.6
POLAB Mix	100 *	2.33 ± 0.20	1.33 ± 0.23	3.96	5.82	4.28	1.4
CTD101K+20% DY040	100 *	2.43 ± 0.27	1.21 ± 0.28	4.39	5.31	4.74	1.5
CEA mix	100 *	2.29 ± 0.69	1.40 ± 0.64	4.02	5.16	5.02	1.3
MSUT	100 *	2.44 ± 0.48	1.34 ± 0.43	3.97	5.33	4.75	1.5
Araldite F	100 *	2.44 ± 0.72	1.89 ± 0.50	3.87	5.43	4.65	1.5
MY750	100 *	4.62 ± 0.56	4.28 ± 1.08	3.94	5.29	4.75	5.3
PMMA	100 *	4.68 ± 0.19	3.90 ± 0.45	3.69	5.49	4.59	5.5
PC	100 *	6.81 ± 0.26	12.2 ± 0.79	3.87	5.61	4.57	12 ^#^
Polyurethane	100 *	7.01 ± 1.29	8.72 ± 1.95	3.96	5.83	4.24	12 ^#^

## Data Availability

Data are contained within the article.
